# Proteinuria Is Associated with Quality of Life and Depression in Adults with Primary Glomerulopathy and Preserved Renal Function

**DOI:** 10.1371/journal.pone.0037763

**Published:** 2012-05-25

**Authors:** Alexandre Braga Libório, João Paulo Lima Santos, Natália Feitosa Arraes Minete, Cecília de Alencar Diógenes, Ariane Pontes Soares, Anaiara Lucena Queiroz, Dulce Maria Silva Barreto

**Affiliations:** 1 Post-Graduate Program in Public Health, UNIFOR, Fortaleza, Ceará, Brazil; 2 Medical Course, University of Fortaleza, UNIFOR, Fortaleza, Ceará, Brazil; 3 Hospital Geral de Fortaleza, Fortaleza, Ceará, Brazil; University of Tokushima, Japan

## Abstract

**Background:**

There is no information about HRQoL, depression and associated factors in adult with nephrotic syndrome-associated glomerulopathy.

**Methodology/Principal Findings:**

Patients with primary glomerulopathy where compared with age and sex-matched hemodialysis patients and healthy subjects. Laboratory data, medical history, comorbid conditions were collected to evaluate factors associated with HRQoL (SF-36) and Depression (Hamilton Depression Rating Scale - HAMD). Glomerulopathy patients had low HRQoL in all eight SF-36 domains and two composite scores (physical and mental) in comparison with healthy subjects. HAMD score also was elevated and there was high depression prevalence. Overall, these data were comparable between glomerulopathy and hemodialysis patients. Using multiple regression analysis, factors associated with low HRQoL physical composite score were: last 24 h-urine protein excretion (−0.183, 95%CI −0.223 to −0.710 for each gram of proteinuria, p = 0.01) and cyclosporine use (−15.315, 95%CI −25.913 to −2.717, p = 0.03). Low HRQoL mental composite score was associated with last 24 h-urine protein excretion (−0.157, 95%CI −0.278 to −0.310 for each gram of proteinuria, p = 0.03) and HMAD score was independently associated with age (0.155, 95%CI 0.318 to 0.988 for each year, p = 0.04), female sex (4.788, 95%CI 1.005 to 8.620, 0 = 0.03), disease duration (0.074, 95%CI 0.021 to 0.128 for each month, p = 0.01) and last 24 h-urine protein excretion (0.050, 95%CI 0.018 to 0.085 for each gram of proteinuria, p = 0.02).

**Conclusions/Significance:**

Nephrotic-syndrome associated glomerulopathy patients have low HRQoL and high prevalence of depression symptoms, comparable with those of hemodialysis patients. Last 24 h-protein excretion rate is independently associated with physical and mental HRQoL domains in addition to depression.

## Introduction

Glomerulopathy is a group of diseases that affect mainly young adults between 20–40 years old [1]. Patients with nephrotic syndrome generally present with important edema, lipid alterations, hypoalbuminemia, and possible loss of renal function. Moreover, treatment involves dietary restrictions and immunosuppression therapy with their adverse effects. All these factors can potentially affect health-related quality of life (HRQoL). Many nephrotic syndrome features are related to a worse HRQoL: protein-nutrition alteration [2], lipid metabolism alteration [3] and reduced glomerular filtration rate (GRF) [4].

Depression is common in general medical patients, with female prevalence of 5 to 9% and 2 to 3% in men [5]. Depression is thought to be associated with worse general health status [6] and is associated with several chronic diseases [7], [8], including chronic Kidney disease (CKD) [9]. In some populations, screening in some special population, such as those undergoing renal replacement therapy, is warranted given its high prevalence and association with increased mortality and morbidity [10]. A close relationship between HRQoL and depression has been well established [11].

Although, it is already well known that CKD is associated with a decreased HRQoL, especially in dialysis populations [4], [12], [13], only recently, adult patients with glomerular disease were studied regarding their HRQoL [14]. Gipson et al. described these patients had a low HRQoL, comparable with dialysis patients [14]. However, this study population was a mix of children and adults with the same glomerular pathology (focal segmental glomerulosclerosis - FSGS) and they were studied at baseline during a therapeutic randomized trial. It was not possible to extrapolate the conclusions to other primary glomerulopathies and there is no information on treatment effects and factors associated with low HRQoL. Moreover, to the best of our knowledge, there is no data about depression in adult glomerulopathy patients.

The objective of the present study is to evaluate HRQoL and depression in a cohort of patients with a diagnosis of nephrotic syndrome-related primary glomerulopathy not undergoing chronic renal replacement therapy.

## Methods

This is a cross-sectional study with patients presenting nephrotic syndrome at the time of a kidney biopsy in a reference university hospital of Brazil (Hospital Geral de Fortaleza). Nephrotic syndrome was defined as 24 h-urine protein greater than 3.5 g/1.73 m^2^ associated with edema, hypoalbuminemia (less than 3.0 g/dL) and lipid alteration. Only patients presenting primary minimal lesions (ML), FSGS, Membranous Nephropathy (MN), IgA nephropathy (IgAN) and membranoproliferative glomerulonephritis (MPGN) at kidney biopsy were considered. Patients with any evidence of secondary glomerulopathy or advanced CKD (GFR less than 15 mL/min/1.73 m^2^) were excluded.

Two age and sex-matched controls groups were selected: one consisting of patients undergoing maintenance hemodialysis for more than three months with no primary glomerulopathy diagnosis and another with healthy subjects. The study protocol was reviewed and approved by the Ethical Committee of the Institution (University of Fortaleza – UNIFOR) and informed consent was obtained from participants.

### Measurements

Subjects underwent structured interviews during a research clinic visit. The interviews were used to obtain patient symptom checklist, medical comorbidities, sociodemographic characteristics, HRQoL and depression assessment. The Short Form-36 (SF-36) questionnaire was applied in the Portuguese version validated in Brazil [15]. This scale covers eight domains, four physical and four emotional ones. Each domain has a scale of 0 to 100, with a higher score always representing better HRQoL. These scales are aggregated into a physical composite score (PCS) and a mental composite score (MCS).

Depression was evaluated by the 17-item Hamilton depression Rating Scale (HAMD) also validated in Brazil [16]. The questionnaire was administered according to the Interview Guide for the Hamilton Rating Scale, of which purpose is to standardize the questions. The questionnaire has a scale that classifies degrees of depression according to these criteria: score lower than 6 – without mood swings/normal; from 7 to 17 – slightly depressed; from 18 to 24 – moderately depressed; above 25 – seriously depressed.

### Treatment Protocol Summary

All patients were treated with angiotensin-converting enzyme inhibitors (ACE inhibitor) or AT1- receptor blocker (AT1R-blocker) when there was no contraindication. In ML and FSGS, the primary treatment was steroid-based therapy and cyclosporine (CsA) when it was steroid-resistant or dependent. MN was treated primarily with CsA or steroids plus cyclophosphamide according to baseline renal function. IgAN presenting with nephrotic syndrome was treated preferentially with steroids and MPGN patients received no immunosuppressive treatment.

### Laboratory Data and Definitions

Medical records were retrieved to assess laboratory data at the kidney biopsy time and the last evaluation prior to study inclusion. Laboratory data included an assessment of serum creatinine, total blood count, serum albumin, total cholesterol, LDL cholesterol, HDL cholesterol, triglycerides, 24 h-urine protein excretion rate (24 h-proteinuria) and urinalysis. Immunosuppressive treatment was considered only when it was taken for at least one month. Edema was considered when it was present in the last clinical evaluation. Hypertension was defined as blood pressure above 140×90 mmHg at three or more medical evaluation or a positive high blood pressure history under regular treatment. Hematuria was considered as more than five red blood cells per high-power microscopic field in two urine samples. Total remission was considered when 24 h-proteinuria was less than 500 mg and partial remission when there was a reduction greater than 50% of initial 24 h-proteinuria and it was less than 3.5 g/24 h/1.73 m^2^. Relapses were considered in patients presenting with 24 h-proteinuria greater than 3.5 g/24 h/1.73 m^2^ and serum albumin less than 3.0 g/dL after achieving partial or total remission. Frequent relapses were considered when there was more than 2 relapses in six months or 3 in a year. Estimated GFR (eGFR) was calculated using simplified MDRD equation. The local ethic's committee approved the study.

### Statistical Analysis

Descriptive statistics are expressed as mean±SD or absolute numbers as appropriate. All variables were tested for normal distribution using the Kolmogorov-Smirnov test. One-way ANOVA with Bonferroni post-test or student's *t*-test was applied to compare means of continuous variables and normal distribution data. Categorical data were tested using the chi-square test.

Continuous estimated predictors of PCS, MCS and HAMD scores included: age, time of disease, body mass index (BMI), eGFR, 24 h-proteinuria at diagnosis, last 24 h-prot, hematocrit, last serum albumin, total cholesterol, LDL cholesterol, HDL cholesterol, triglycerides. Spearman's correlation coefficients (*r*_s_) were used to determine whether these estimated predictors correlated with PCS, MCS and HAMD scores.

Patients were categorized into groups based on gender, presence of diabetes mellitus, blood pressure status (normotensive *versus* hypertensive), use of ACE inhibitor or AT1R-blocker, statins, steroids, CsA, cyclophosphamide (yes *vs.* no for each drug class), presence of hematuria and remission status (total or partial vs. no-remission). Comparisons of PCS, MCS and HAMD scores between the abovementioned subgroups were performed using a two-sample *t* test.

Subsequent multiple linear regression modeling was used to determine which variables that were significant in the univariate analysis were independently associated with PCS, MCS and HAMD score. Models for PCS, MCS and HAMD scores were determined separately. Corresponding interaction terms were included in the initial model. Backwards elimination was performed, and variables (interaction terms and predictors) that were nonsignificant at the α = 0.05 level were sequentially removed from the model. Stepwise selection was also performed in an interactive manner, where variables with a p-value<0.10 were added to the model and variables with a p-value>0.05 were removed. The statistical analysis was performed using SPSS 19.0 for windows. *p* values<0.05 were considered as statistically significant.

## Results

### Population Characterization

From a group of 311 patients, 135 nephrotic-associated glomerulopathy patients were pre-selected and an interview was conducted in 99 glomerulopathy patients. Contact was not possible with other 36 patients. Mean age was 36±11 years old. Ninety-nine hemodialysis patients and healthy subjects age- and sex-matched were selected. Mean time of dialysis was 38±16 months.

Complete data on clinical and demographic characteristics of glomerulopathy patients are shown in table 1. Main histological diagnosis was FSGS (n = 49), MN (n = 29), ML (n = 4). Forty-three patients were receiving steroid drugs and nineteen received cyclosporine-based therapy. Patients undergoing steroid therapy were receiving a mean dose of 0.2 mg/Kg/day of prednisone. Thirty-eight patients were in total and 26 in partial remission. Thirty-five patients had achieved no remission. Twenty-six patients had had at least one relapse in the last three years before study entry and the mean of relapses frequency in these patients was 0.7/year. Only 3 patients were considered as having frequent relapses.

**Table 1:** Patients' clinical and demographic characteristics. Data are presented as mean±SD or absolute values.

	Glomerulopathy patients (n = 99)
Age (years)	36±11
Gender (M/F)	46/53
Renal biopsy diagnosis	
*FSGS*	49
*MN*	29
*IgAN*	9
*MPGN*	8
*ML*	4
Disease duration (months)	33.6±26.0
Arterial Hypertension	40
Diabetes	6
Familial nephropathy history	21
Presence of edema	31
Mean eGFR (mL/min/1.73 m^2^)	61±29
CKD stage	
*stage 1*	55
*stage 2*	24
*stage 3*	16
*stage 4*	04
ACE inhibitor/AT1R blocker	62
Steroid therapy	43
Cyclosporine therapy	19
Cyclophosphamide therapy	03
Hemoglobin (g/dL)	12.3±0.9
Last serum albumin (g/dL)	3.6±0.9
Last total cholesterol (mg/dL)	221±98
Last triglycerides (mg/dL)	146±58
Proteinuria at diagnosis (g/24 h/1.73 m^2^)	8.6±3.8
Last proteinuria (g/24 h/1.73 m^2^)	2.8±2.2
Remission	
Total	38
Parcial	26
No	35

FSGS: focal and segmentar glomerulosclerosis; ML: minimal lesions; MN: membranous Nephropathy; IgAN: IgA nephropathy; MPGN: membranoproliferative Glomerulonephritis; CKD: Chronic Kidney Disease; ACE: angiotensin converting enzyme; AT1R: angiotensin 1 receptor.

### Quality of Life and Depression in Glomerulopathy Patients

Mean scores in each SF-36 domain in healthy, dialysis and glomerulopathy patients are shown in table 2. As expected, dialysis patients had a low score in all domains, when compared to healthy subjects, except in mental health. Glomerulopathy patients had low scores in all eight domains, when compared with healthy subjects. Glomerular patients had better scores at the physical functioning scale and worse mental health than hemodialysis patients. All other domains were similar between these two groups. Mental and physical health composite scores were lower in both dialysis and glomerulopathy patients than in healthy subjects was observed no difference between these two last groups.

**Table 2:** SF-36 HRQoL and HAMD comparison between glomerulopathy and hemodialysis patients and healthy subjects.

	Glomerulopathy patients (n = 99)	Hemodialysis patients (n = 99)	Healthy subjects (n = 99)
Physical functioning scale #	70.4±27.0	52.7±34.1	91.4±19.3
Role, physical*	37.9±22.5	26.7±20.9	87.0±31.2
General Health*	63.2±34.8	54.3±24.9	81.8±24.9
Bodily pain*	71.8±28.4	73.7±26.9	90.9±21.2
Mental Health**	66.5±22.6	78.3±19.8	78.4±18.4
Role, emotional*	57.4±36.22	70.0±35.4	97.3±14.8
Vitality*	62.2±22.1	63.1±26.5	73.7±19.1
Social functioning*	64.3±34.0	69.6±33.1	92.5±19.2
Physical health composite score*	61.6±21.1	56.6±21.4	85.7±14.9
Mental health composite score*	62.9±31.9	68.1±21.9	87.5±14.3
Hamilton depression Rating Scale∂	8.3±6.9	7.0±5.7	5.1±4.1

#p<0.001 between all groups,

* p<0.001 between healthy subjects and others groups,

** p<0.01 between glomerulopathy patients and others groups,

∂ p = 0.01 between glomerulopathy patients vs. healthy subjects.

Glomerulopathy patients were more depressed according to HAMD than healthy subjects (48 vs. 22.4%, p = 0.002) and were similar to dialysis patients (48 vs. 43%, p = 0.302). No patient was receiving depression therapy. Overall, glomerulopathy and dialysis patients had comparable HAMD scores (8.3±6.9 vs. 7.0±5.7, p = 0.613) and glomerulopathy patients had higher scores than healthy patients (5.1±4.1, p = 0.01) – see table 2. HAMD scores presented a significant inverse correlation with mental and physical health composite scores (r = −0.402 and −0.391, p<0.001 for both). There was no difference between patients according to histological diagnosis (data not shown).

### Factors Associated with Quality of Life and Depression in Glomerulopathy Patients

Univariate analysis comparing the potential contributors to PCS, MCS and HAMD score were evaluated (tables 3 and 4). Using variables associated with each score, multiple regression analysis demonstrated that last 24 h-proteinuria and CsA use were associated with PCS; last 24 h-proteinuria was the only factor associated with MCS and gender, age, last 24 h-proteinuria and disease duration were independently associated with HAMD scores (table 5). Proteinuria at diagnosis (*r*_s_ = −0.064, p = 0.532 with PCS; −0.016, p = 0.874 with MCS and 0.016, p = 0.888 with HAMD), eGFR (*r*_s_ = 0.049, p = 0.613 with PCS; 0.078, p = 0.322 with MCS and 0.061, p = 0.496 with HAMD), mean daily prednisone dose in the last 6 months ((*r*_s_ = −0.031, p = 0.723 with PCS; 0.012, p = 0.822 with MCS and 0.022, p = 0.896 with HAMD) and partial or total remission were not associated with any score evaluated.

**Table 3:** Scores in subgroups based on several clinical characteristics.

Characteristic	Score	Score	p
Gender	Male (n = 46)		
PCS	64.3±19.7		0.225
MCS	63.7±19.9		0.242
HAMD	6.3±4.7		0.010
Hypertension	yes (n = 48)	no (n = 51)	
PCS	58.4±20.3	63.2±21.0	0.302
MCS	58.9±19.4	61.7±22.3	0.568
HAMD	9.3±6.5	7.9±6.5	0.388
Diabetes Mellitus	yes (n = 5)	no (n = 84)	
PCS	56.4±17.3	61.6±21.0	0.589
MCS	64.9±16.6	60.3±21.4	0.641
HAMD	8.0±6.6	b8.5±6.2	0.883
ACE inhibitor/AT1R-blocker	yes (n = 68)	no (n = 31)	
PCS	59.0±19.8	63.8±22.7	0.325
MCS	58.8±20.6	63.4±21.8	0.352
HAMD	8.3±6.5	8.7±6.3	0.844
Statin	yes (n = 21)	no (n = 78)	
PCS	54.6±21.4	63.1±20.4	0.084
MCS	56.4±21.1	62.0±21.0	0.298
HAMD	8.3±6.3	8.6±5.8	0.891
Steroid therapy	Yes (n = 66)	no (n = 33)	
PCS	58.0±18.9	62.8±22.16	0.310
MCS	58.9±20.12	61.7±21.8	0.562
HAMD	7.2±6.1	9.3±7.7	0.093
CsA therapy	yes (n = 19)	no (n = 80)	
PCS	53.8±17.8	62.2±21.3	0.035
MCS	51.9±19.8	62.2±21.0	0.045
HAMD	10.4±8.9	8.1±6.9	0.203
Cyclophosphamide therapy	yes (n = 6)	no (n = 93)	
PCS	54.7±12.9	61.1±21.4	0.552
MCS	65.6±17.2	60.4±21.4	0.634
HAMD	6.2±5.2	8.8±6.9	0.321
Hematuria	yes (n = 27)	no (n = 72)	
PCS	55.2±17.4	65.4±21.7	0.042
MCS	56.2±21.6	65.2±19,7	0.071
HAMD	7.5±6.1	8.4±6.2	0.590
Remission	yes (n = 64)	no (n = 35)	
PCS	61.5±19.9	60.7±27.2	0.906
MCS	61.7±19.1	61.4±27.7	0.961
HAMD	8.6±6.6	7.9±6.2	0.477
Relapses at last three years	yes (n = 26)	no (n = 73)	
PCS	59.7±16.8	61.7±19.6	0.771
MCS	60.3±18.0	62.0±21.6	0.693
HAMD	8.2±6.2	8.4±6.2	0.931

PCS: SF-36 Physical composite score; MCS: SF-36 Mental composite score; HAMD: Hamilton Depression Rating Scale; CsA: cyclosporine.

**Table 4:** Significant correlations (*r*_s_) between PCS, MCS and HAMD score with clinical and laboratory data.

	Physical Composite Score	p	Mental Composite Score	p	HAMD Score	p
Age (years)	-	-	-	-	0.200	0.034
Disease duration (months)	-	-	-	-	0.400	0.001
Last 24 h-proteinuria (mg)	−0.233	0.024	−0.237	0.019	0.223	0.029
Last cholesterol, total (mg/dL)	-	-	−0.208	0.029	0.250	0.012

**Table 5:** Variables independently associated with PCS, MCS and HAMD score.

Physical Composite Score			
*Variable*	*B*	*95%CI for B*	*p*
Last 24 h-proteinuria (g/24 h)	−0.182	−0.223 to −0.710	0.01
CsA use	−15.315	−25.913 to −2.717	0.03

Mental Composite Score			
*Variable*	*B*	*95%CI for B*	*p*
Last 24 h-proteinuria (g/24 h)	−0.157	−0.278 to −0.310	0.03

HAMD score			
*Variable*	*B*	*95%CI for B*	*p*
Gender (female)	4.788	1.005 to 8.620	0.03
Age (years)	0.155	0.318 to 0.988	0.04
Disease duration (months)	0.074	0.021 to 0.128	0.01
Last 24 h-proteinuria (g/24 h)	0.050	0.018 to 0.085	0.02

## Discussion

In this study, adult glomerulopathy patients had worse HRQoL and high depression prevalence and severity than healthy subjects, being comparable with patients on maintenance hemodialysis. Moreover, we determined that actual proteinuria was associated with HRQoL and depression.

Glomerulopathy patients have several characteristics that can affect quality of life. Presence of severe edema, loss of renal function, daily activities and diet restriction can potentially reduce physical and mental aspects of quality of life. Moreover, treatment-related adverse effects can be associated with poor quality of life. However, only recently it has been reported that FSGS patients with no treatment had low HRQoL scores [14], but there is no information about the factors associated with this low HRQoL and if reduction-proteinuria treatment has any impact on its improvement. Moreover, depression was never studied in glomerulopathy patients before.

It is known that advanced CKD patients have poor HRQoL and severe depression diagnosis, whether they are undergoing or not renal replacement therapy. [13], [17]–[19] In our data, glomerulopathy patients had low HRQoL and depression, comparable to hemodialysis patients. Of eight SF-36 domains, six showed similar scores between glomerulopathy and hemodialysis patients. Only functional capacity was worse in hemodialysis patients; similar findings were observed by Gipson et al. [14], when evaluating FSGS patients. Although HRQoL in hemodialysis patients is extensively described in literature [4], we prefer to reassess these data with an age- and sex-matched group, due to possible regional differences when comparing reports from developed countries.

Abdel-Kader et al. described patients with stage 4 and 5 CKD that had comparably low HRQoL and depression [19]; however, our cohort had a mean eGFR of 61±29 mL/min/1.73 m^2^ and only six patients had stage 4 CKD. It is unlikely that low HRQoL and depression burden in glomerulopathy patients be due only to a reduced eGFR.

Only three populations with glomerulopathy have been studied regarding HRQoL: pediatric patients with steroid-sensitive nephrotic syndrome [20], children and adults with untreated FSGS [14]. These studies have demonstrated a low HRQoL in nephrotic patients. Our data includes several histological glomerulopathies types. This is important in data validation and because different features of the glomerulopathy type (remission index and CKD evolution) can have different impacts on HRQoL.

To the best of our knowledge, depression has not been assessed in glomerulopathy patients. In this study, glomerulopathy patients had higher HAMD score than healthy subject and were comparable to hemodialysis patients. In the general population, prevalence of depression in women ranges from 5 to 9% and 2 to 3% in men [5]. It is important to distinguish between the diagnosis of major depressive disorder and the symptoms of depression [9], with the first being evaluated by DSM-IV [21]. HAMD is used as an instrument to screen for depression, so it is not possible to estimate the true prevalence of depression in the studied population. In spite of these considerations, glomerulopathy patients presented high HAMD scores and depression must be assessed by a physician in this population.

It is unknown if glomerulopathy-directed treatment can ameliorates HRQoL and depressive symptoms. An ongoing randomized clinical trial is using HRQoL as an end-point in FSGS patients [14]. In our sample, patients achieving partial or total remission had comparable scores for both HRQoL and HAMD when compared with those not achieving remission. However, the presence of 24 h-proteinuria was independently associated with PCS, MCS and HAMD score. These data suggest that proteinuria reduction, even when not sufficient to be classified as remission, can be important in order to improve HRQoL and depression symptoms. This is important because using remission criteria when evaluating HRQoL and depression in glomerulopathy patients can be inaccurate.

It is not possible to determine the pathophysiological background of the association between proteinuria and quality of life and depression. It is possible that subjective factors such as concerns about disease control and prognosis may interfere directly in the assessment of HRQoL. Also, patients with higher proteinuria levels have more dietary and physical activity restrictions. Moreover, proteinuria is associated with inflammation [22]. In other medical conditions [23], [24], inflammation has been associated with depression and HRQoL, suggesting that future studies must explore this possible relationship.

Cyclosporine use was associated with low PCS. This information is dissimilar from other data in renal transplantation [25], where steroid-therapy is associated with low physical performance. In our study, less than half of the patients were receiving steroid therapy (n = 43), with a mean dose of 0.2 mg/Kg/day. In glomerulopathy, steroid therapy is generally limited to a six-month course and steroid-resistant or dependent patients are generally changed to CsA-based therapy. This short-term of steroid therapy, in contrast with other clinical conditions, i.e. renal transplantation, can explain the absence of association between steroid dose and HRQoL and HAMD scores.

In our patients, cyclosporine was used as primary choice in MN and in steroid-resistant or dependent FSGS and ML. Generally, these patients maintain CsA use for one year after achieving remission, in contrast with steroid-treated patients that generally undergo a 4 to 6 month-duration therapy. We speculate this long-course CsA therapy can adversely affect the physical component. Moreover, two patients receiving CsA had moderate/severe depressive symptoms. Depression has been associated with CsA therapy [26] and it is reversible after its withdrawal. Although no data was available about depression diagnosis before CsA initiation in our patients, CsA must be considered as having an etiological role.

Disease duration and age were associated with depression. It is known that many chronic medical conditions can impair HRQoL and increase depression prevalence [7], [27]. It is known there is a higher prevalence of depression in older subjects [28]. As in other chronic diseases [29], [30], in this sample disease duration was also associated with depression symptoms.

Renal function and protein excretion rate at diagnosis were not associated with any score. The first can be explained due to high eGFR in our sample, where 60% had only mild CKD (eGFR>60 mL/min/1.73 m^2^). The lack of association between 24 h-proteinuria at diagnosis and the association with the present one highlights the importance of proteinuria reduction in ameliorating HRQoL and depression.

Our study has several limitations. First, it was performed in a developing country. The results can be different in developed countries. In the present study, we evaluated a relatively small number of hemodialysis patients, but our data is comparable with those in literature [14], making this a valid control group. Moreover control groups were age- and sex-matched. Lastly, we cannot explain the exact mechanism of the association between 24 h-proteinuria and HRQoL and depression.

In conclusion, adult nephrotic syndrome-related glomerulopathy patients had low HRQoL and high depression symptom scores. These were comparable with those in hemodialysis patients. Presence of proteinuria was associated with worse HRQoL and depression. We suggest that classical cutoff points of urine protein excretion to classify remission are not sensitive to evaluate amelioration in HRQoL and depression symptoms.
